# To the Editors of the Medical and Physical Journal

**Published:** 1802-04

**Authors:** 


### *To the Editors of the Medical and Phyſical Journal.*

GENTLEMEN,

MR. Cooke's practice of waſhing patients affected with contagious fever, as well as perſons expoſed to the riſk of contagion, with vinegar, cannot be too generally adopted. I have no doubt it will give him much pleaſure to be informed that a ſimilar mode of treatment was followed, with the moſt marked ſucceſs, in the plague which occaſioned ſo much havoc in Ruſſia, in the year 1771. In various parts of his Treatiſe, De Mertens adviſes ablution with vinegar; and in his account of the means by which he kept the great Foundling Hoſpital at Moſcow free from the peſtilence, he particularly mentions that he directed the "children who were brought (from infected families) to the quarantine-houſe, to be ſtripped to the ſkin; after which their clothes were burnt, *their bodies waſhed all over with vinegar and water*, and new clothes put upon them."

*March* 13, 1802. ANTILOIMICUS.

